# Ultrathin dual-mode vortex beam generator based on anisotropic coding metasurface

**DOI:** 10.1038/s41598-021-85374-4

**Published:** 2021-03-11

**Authors:** Liang Zhang, Jie Guo, Tongyu Ding

**Affiliations:** School of Information Engineering, Jimei University, Xiamen, 361021 China

**Keywords:** Applied physics, Metamaterials

## Abstract

In this paper, an anisotropic coding metasurface is proposed to achieve dual-mode vortex beam generator by independently manipulating the orthogonally linearly polarized waves. The metasurface is composed of ultrathin single-layer ground-backed Jerusalem cross structure, which can provide complete and independent control of the orthogonally linearly polarized incident waves with greatly simplified design process. As proof of concept, a metasurface is designed to generate vortex beams with different topological charges under orthogonal polarizations operating at 15 GHz. Experimental measurements performed on fabricated prototype reveal high quality, and show good agreements with theoretical designs and simulation results. Such ultrathin dual-mode vortex beam generator may find potential applications in wireless communication systems in microwave region.

## Introduction

As one of the natural properties of electromagnetic (EM) waves, angular momentum plays an essential role in the manipulation of EM waves including spin angular momentum (SAM) and orbit angular momentum (OAM). For SAM, it is revealed by Poynting that circularly polarized light should have an angular momentum, and related with the polarization state of the light^1^. Compared with SAM, light carrying OAM are characterized by a helical phase of exp(i*lθ*), where *l* is the topological charge and *θ* is the azimuthal angle^2^. The topological charge *l* can take any integer values, which are orthogonal and independent with each other. Due to this unique property, individual channels can be built based on different OAM modes with little interaction on each other, leading to potential of information multiplexing^3^. Since discovered in 1992 by Allen et al.^2^, vortex beam which carrries OAM has been widely applied in visible spectrum, such as optical manipulation, optical trapping, imaging and so on^4–7^. Recent studies imply that OAM can be introduced to radio region as well^8–10^. In 2007, Thid et al. first proposed that the vortex beam can be generated by phased-array antennas, opening the door to apply OAM in radio frequency domain^3^. Later in 2011, Tamburini et al. confirmed the feasibility and validity of the vortex beam for wireless communication, and the vortex beams were generated and detected in laboratory in radio frequency^8^. In 2012, Tamburini et al. proposed an OAM-based wireless communication system over a distance of 442 m to encode two radio waves with vortex beams with different topological charges at same frequency^9^. Vortex beam was further applied in millimeter-wave domain to achieve as dual-channel 60 GHz communications, where 8-Gbit/s and 4-Gbit/s wireless link were realized using different OAM modes^10^.

For vortex beam generation, spiral phase plate^11^ and holographic diffraction gratings^12^ are widely used in visible and terahertz region. For radio frequency, vortex beams are usually generated by spiral reflectors^9,13^ and antenna arrays^3,14^. However, the spiral reflector suffers from manufacturing complicity due to its specially cured surface, and is difficult to be integrated with other equipment. For antenna arrays, complex feeding network is a must to achieve the desired phase difference between array elements. More recently, metasurfaces are reported to manipulate electromagnetic waves of independent control of phase, amplitude and polarization^15–17^, and have led numerous developments of practical applications such as flat lens^18,19^, retroreflectors^20,21^, hologram^22–25^, multifunctional devices^26–28^, to name a few. Following the design procedure of planar arrays, metasurfaces have been designed to generate vortex beams carrying OAM in optical and radio frequency^29–40^. Compared to traditional spiral reflectors and antenna arrays, metasurfaces not only show advantages of low profile, small mass, low manufacturing cost, and ease of deployment, but also establish the feasibility of mode multiplexing of OAM beams. In general, there are several basic techniques for OAM mode multiplexing, including multiband^34^, direction^35^ and polarization^36–38^. In 2018, Meng et al. proposed dual-band reflectarray for generating dual beams with respect to carrying two different OAM topological charges operating in different bands^34^. Also in 2018, Meng et al. proposed an anisotropic holographic metasurface to generate multiple beams with different orbital angular momentum (OAM) modes in different directions^35^. Polarization multiplexing relies on the use of polarization state as an additional degree of freedom. Previously published works have reported the implementation of the dual-polarization dual-mode OAM generator with two orthogonal states of linear polarization in transmission or reflection mode. Multi-layer structures were utilized as the unit cell to achieve independent control of the transmitted TE and TM waves^36,37^. Such multi-layer structures usually suffer from high profile and fabrication cost. In 2016, Yu et al. proposed a reflective metasurface to generate OAM waves of dual polarizations and dual modes in the radio frequency domain with single-layer structure^38^. However, the air layer between the substrate and the metallic ground increases the difficulty in installation and integration in system.

In this paper, we further demonstrate the simultaneous generation of dual-mode convergent OAM vortex beams utilizing a reflective metasurface under orthogonally linearly polarized incidences. Compared with previous published works, the proposed metasurface not only show the ultrathin property, but also use only single layer substrate without air gap between the substrate and the metal ground, which provides more advantages in fabrication, installation and integration. Such dual-mode functionality provided by a single metasurface shows advantage in the multiread communication system to replace two independent OAM antennas with orthogonal polarizations. The proposed metasurface is constructed by ground-backed Jerusalem cross elements, which enable to independently manipulate the orthogonal linear polarizations. By elaborately designing the arm lengths of the Jerusalem cross elements, a group of 16 digital elements (“00 + 00” to “11 + 11”) is extracted to construct the metasurface. The digits before and after the “ + ” indicate the operation status of the element under *x*- and *y*-polarized incidences, and “00”, “01”, “10” and “11” correspond to the reflected phase response of “0”, “π/2”, “π” and “3π/2”, respectively^41,42^. Then the proposed metasurface is formed by arranging the digital elements based on the coding maps discretized from the calculated phase distribution. The simulated and experimental results verify the effectiveness of the proposed design.

## Results

**Figure 1:**
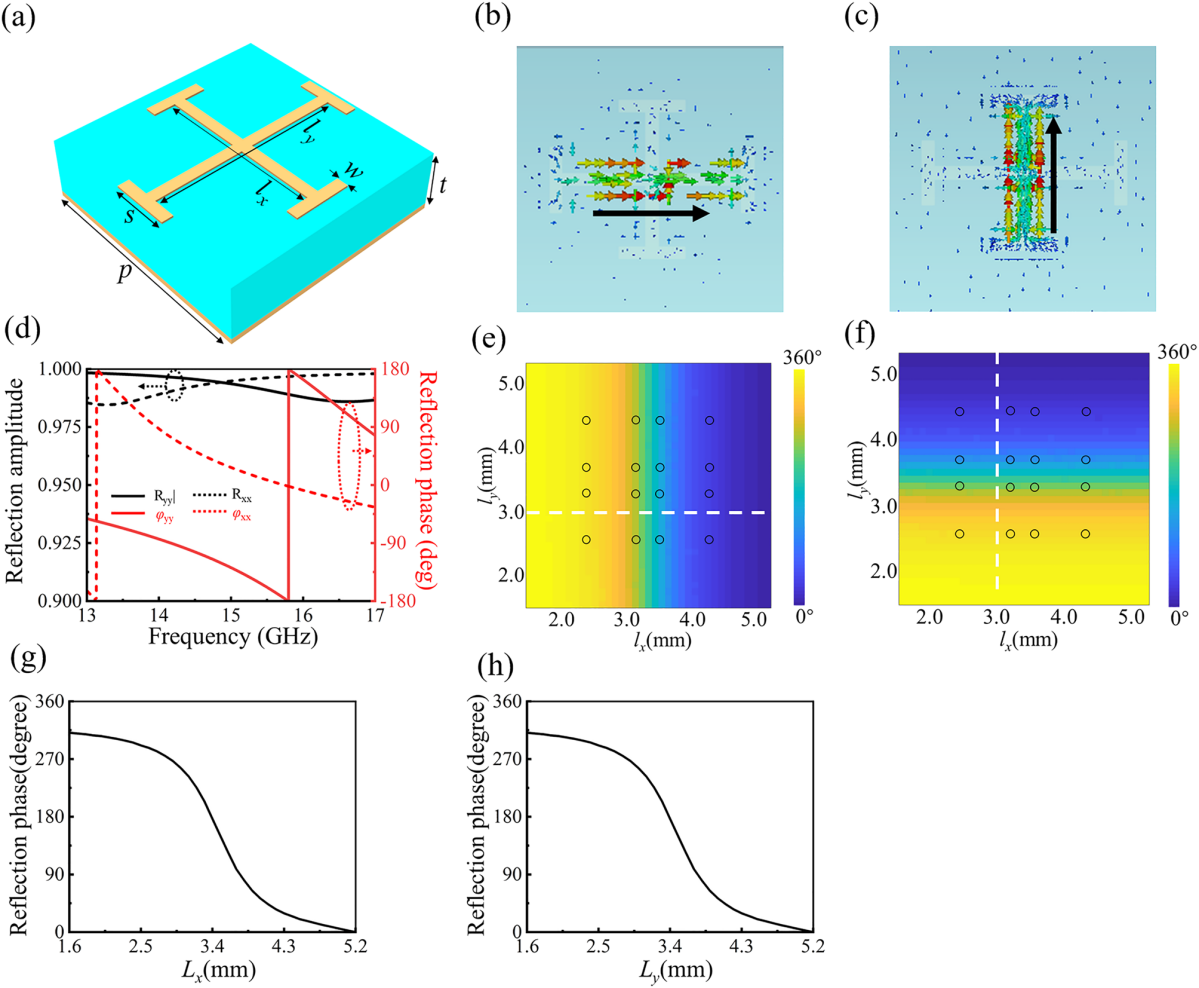
(**a**) Schematic view of the coding element. (**b**, **c**) Current distribution on the top metallic layer of the proposed meta-atom under *x*- and *y*-polarized incidences, respectively. (**d**) Reflection coefficient of the meta-atom with *l*_*x*_ = 4 mm and *l*_*y*_ = 3 mm. (**e**, **f**) Simulated reflection phase response under *x*- and *y*-polarized incidences as functions of *l*_*x*_ and *l*_*y*_, respectively. (**g**, **h**) Reflection phase under *x*- and *y*-polarized incidences corresponding to the white dotted line in (**e**, **f**).

The key step to realize the proposed dual-mode vortex beam generator is to elaborately design meta-atoms which can provide independently control of the orthogonal linear polarizations. A reflective single-layer metallic structure is proposed, in which a metallic Jerusalem cross structure is printed on a ground-backed dielectric substrate with thickness of *t* = 1.5 mm (only 0.075*λ*_*0*_ at 15 GHz), the permittivity of *ε*_*r*_ = 3, and the loss tangent of tan *δ* = 0.0015. Other geometrical parameters shown in Fig. 1a are *p* = 6 mm, *s* = 1.5 mm, *w* = 0.3 mm. *l*_*x*_ and *l*_*y*_ are changed independently to control the reflection phases under *x*- and *y*-polarized incident waves, respectively. The proposed meta-atom is simulated using the commercial software CST Microwave Studio by applying unit periodic boundary conditions in *x*- and *y*-directions. Figure 1b,c show the current distribution of the proposed meta-atom with fixed parameters *l*_*x*_ = 4 mm and *l*_*y*_ = 3 mm under *x*- and *y*-polarized incidences, respectively. It can be observed that only the I-shaped structure in the horizontal direction is resonant when it is illuminated by *x*-polarized wave, while only the I-shaped structure in the vertical direction is resonant when the meta-atom is illuminated by *y*-polarized wave, indicating that *x*- and *y*-polarized waves can be independently manipulated by changing the crossed I-shaped structures. Under the illumination of the linearly polarized wave, the I-structure operates as a dipole antenna. By changing the lengths of *l*_*x*_ or *l*_*y*_, the resonance frequency of I-structure can be modulated, and thus different reflection responses can be obtained. Figure 1d depicts the reflection amplitude and phase spectra of the proposed meta-atom with fixed parameters *l*_*x*_ = 4 mm and *l*_*y*_ = 3 mm under *x*- and *y*-polarized incidences, respectively. The amplitudes of both component (R_xx_ and R_yy_) are above 0.98 at 15 GHz, indicating that almost all the reflected wave is reflected in a co-polarized channel. Figure 1e shows the simulated reflection phase response of *x*-polarized component under *x*-polarized incidence as functions of *l*_*x*_ and *l*_*y*_, from which it can be observed that the reflection phases of the *x*-polarized component keeps unchanged with the variation of *l*_*y*_, and can be changed from 0° to 312° with the variation of *l*_*x*_ (changed from 1.5 mm to 5.2 mm). Similarly, Fig. 1f shows the reflection phase response of *y*-polarized component under *y*-polarized incidence as functions of *l*_*x*_ and *l*_*y*_, from which it can be observed that the reflection phases of the *y*-polarized component can be changed from 0° to 312° with the variation of *l*_*y*_ (changed from 1.5 mm to 5.2 mm), and it keeps unchanged with the variation of *l*_*x*_*.* Hence, it can be concluded that the reflection phases of the *x*- and *y*-polarized waves can be controlled independently by using such Jerusalem cross structure by changing the lengths of *l*_*x*_ and *l*_*y*_, respectively. To highlight the phase coverage, reflection phase is extracted along the white dotted line traced in Fig. 1e,f, and plotted versus *l*_*x*_ (*l*_*y*_) with other parameter *l*_*y*_ (*l*_*x*_) fixed in Fig. 1g,h. It can be seen from Fig. 1g,h that significant phase coverage of 312° is obtained for the dual linear polarizations. Although higher bit level of coding elements can decrease the phase discretization and enhance the performance of the metasurface, the 2-bit coding level is adopted here as a good tradeoff between design complexity and discretization losses. The proposed design method for meta-atoms and holographic metasurfaces can be extended toward higher frequencies, such as the millimeter wave, terahertz (THz) and visible frequency regions, and can also be applied for transmission-type metasurfaces.

**Figure 2:**
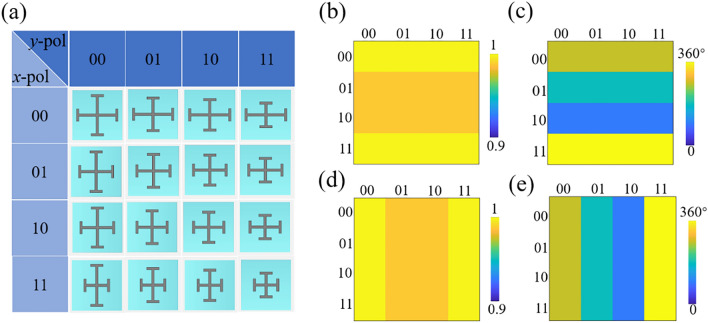
(**a**) Details of the designed 16 coding elements. Phase responses of the coding elements along the horizontal and vertical axes indicate the operational state under *x*- and *y*-polarized incidences. (**b**, **c**) The reflection amplitude and phase responses of the 16 coding elements under *x*-polarized incidence, respectively. (**d**, **e**) The reflection amplitude and phase responses of the 16 coding elements under *y*-polarized incidence, respectively.

Based on the simulated results shown in Fig. 1b,c, 16 coding elements exposing arbitrary 2-bit reflection phase of “0”, “π/2”, “π” and “3π/2” under each *x*- and *y*-polarized incidence are extracted, as marked in Fig. 1e,f. Details of the designed 16 coding elements are shown in Fig. 2a. Phase responses along the horizontal and vertical axes indicate the operational state under *x*- and *y*-polarized incidences of the coding elements. The reflection phase and amplitude of the designed coding elements at 15 GHz are depicted in Fig. 2b–e for *x*- and *y*-polarized incidences, respectively. As discussed above, owing to low dielectric loss of the substrates, negligible absorption occurs under both incidences. Thus the reflection amplitudes of the designed coding elements keep above 0.95, as shown in Fig. 2b,d, guaranteeing the high efficiency of the OAM generator. It can be observed from Fig. 2c,e that the designed coding elements expose independent 2-bit phase state under each *x*- and *y*-polarized incidences, providing the freedom to independently manipulate the orthogonal linear polarizations.

**Figure 3:**
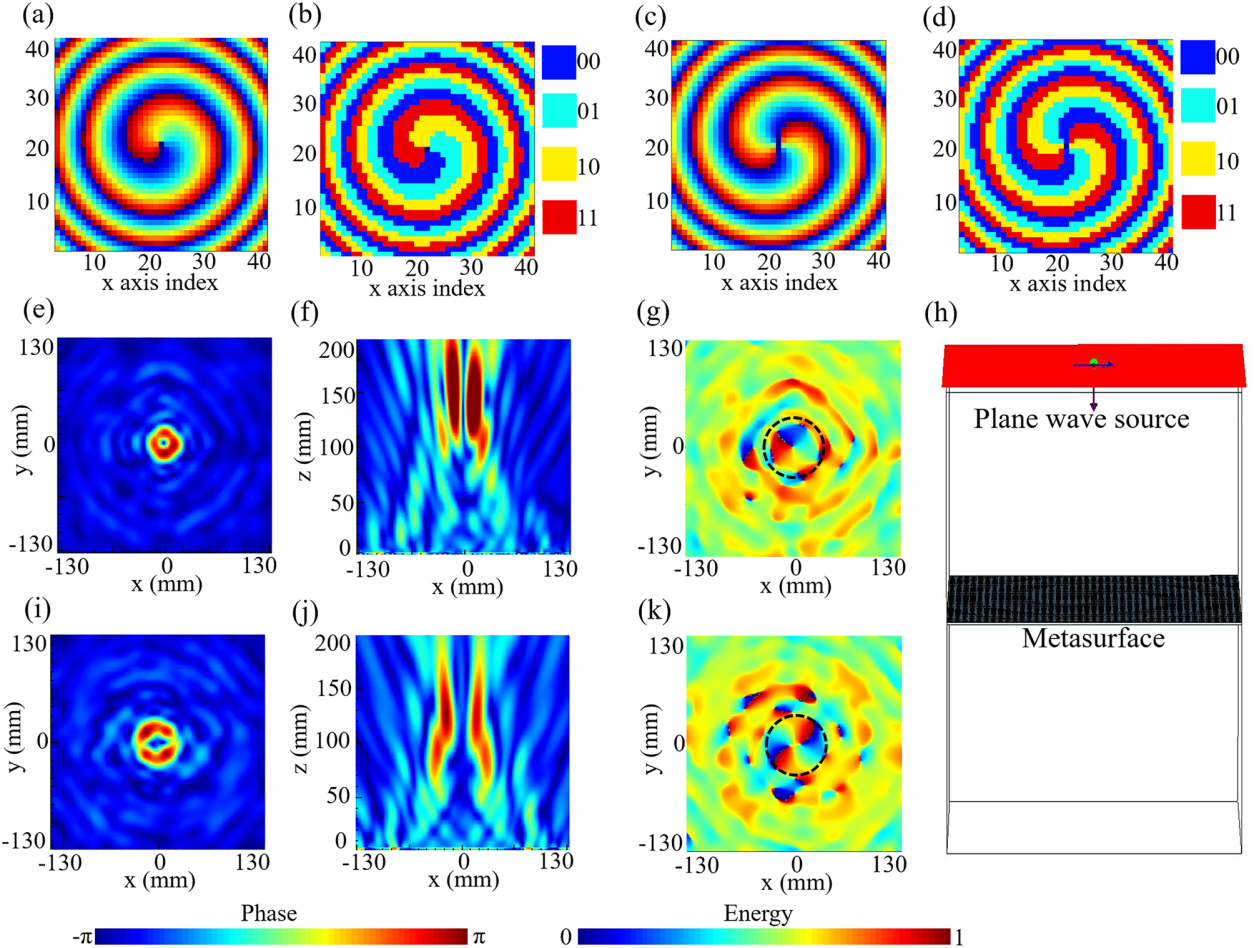
(**a**) Calculated phase distribution and (**b**) discretized code distribution for *x*-polarized incidence. (**c**) Calculated phase distribution and (**d**) discretized code distribution for *y*-polarized incidence. (**e**) Simulated energy distribution at *z* = 150 mm in *xoy* plane and (**f**) the corresponding phase distribution under *x*-polarized incidence. (**g**) Simulated energy distribution in the *xoz* plane under *x*-polarized incidence. (**h**) The simulation model in CST Microwave Studio. (**i**) Simulated energy distribution at *z* = 150 mm in *xoy* plane, and (**j**) the corresponding phase distribution under *y*-polarized incidence. (**k**) Simulated energy distribution in the *xoz* plane under *y*-polarized incidence.

To achieve the converged vortex beams utilizing the proposed reflective metasurface, the theoretical phase compensation *φ* of each meta-atom should satisfy the following equation^32^:

1 $$ \varphi = 2\pi \left( {\sqrt {(x^{2} + y^{2} ) + F^{2} } - F} \right){/}\lambda + l \cdot \arctan (y{/}x) $$

where *l* is topological charge of the desired vortex beam, (*x*, *y*) are the meta-atom position coordinates, *λ* is the free-space wavelength at 15 GHz, and *F* is the focal length which is set to be 7.5*λ* (150 mm). The topological charge *l* is set to be 1 and 2 for *x*- and *y*-polarized incidences, respectively. The calculated phase distributions of *x*- and *y*-polarized incidences are shown in Fig. 3a,c, respectively. Since four phase states are available for the proposed elements, the continuous phase distribution is further quantized to 2-bit level, as shown in Fig. 3b,d. Then, the polarization multiplexed 2-bit vortex beam generator can be constructed by superimposing the two obtained coding maps based on the coding rule of Fig. 2a.

To demonstrate our proposed polarization multiplexed vortex beam generator, the 2-bit coding metasurface is designed and simulated to project vortex beams carrying the OAM mode *l* = 1 and *l* = 2. The metasurface consists of 41 × 41 meta-atoms with an overall size of 246 mm × 246 mm. In the near-field imaging simulations, the metasurface is subjected to open boundary conditions along *x*- and *y*- axes, and is illuminated by *x*- and *y*-polarized waves respectively. Figure 3h shows the whole simulation model. The metasurface is simulated using the commercial software CST Microwave Studio by applying open boundary conditions in *x*-, *y*- directions and open and add space boundary conditions in *z*-direction. The ideal plane wave is used as the feeding source. Figure 3e–g present the vortex beam with *l* = 1 generated by the proposed metasurface under *x*-polarized incidence, while the results of the vortex beam with *l* = 2 under *y*-polarized incidence are exhibited in Fig. 3i–k. Figure 3e,i depict the simulated energy distribution in the *xoy* plane at *z* = 150 mm, where a clear doughnut-shaped energy ring represents the generation of the vortex beam, and the helical phase pattern distribution in Fig. 3f,j can be observed. Figure 3g,k present the simulated energy distribution at a cross section along the propagation direction (*xoz* plane with *y* = 0). The hollow and divergent characteristics further verify the generation of vortex beams under *x*- and *y*-polarized incidences.

**Figure 4:**
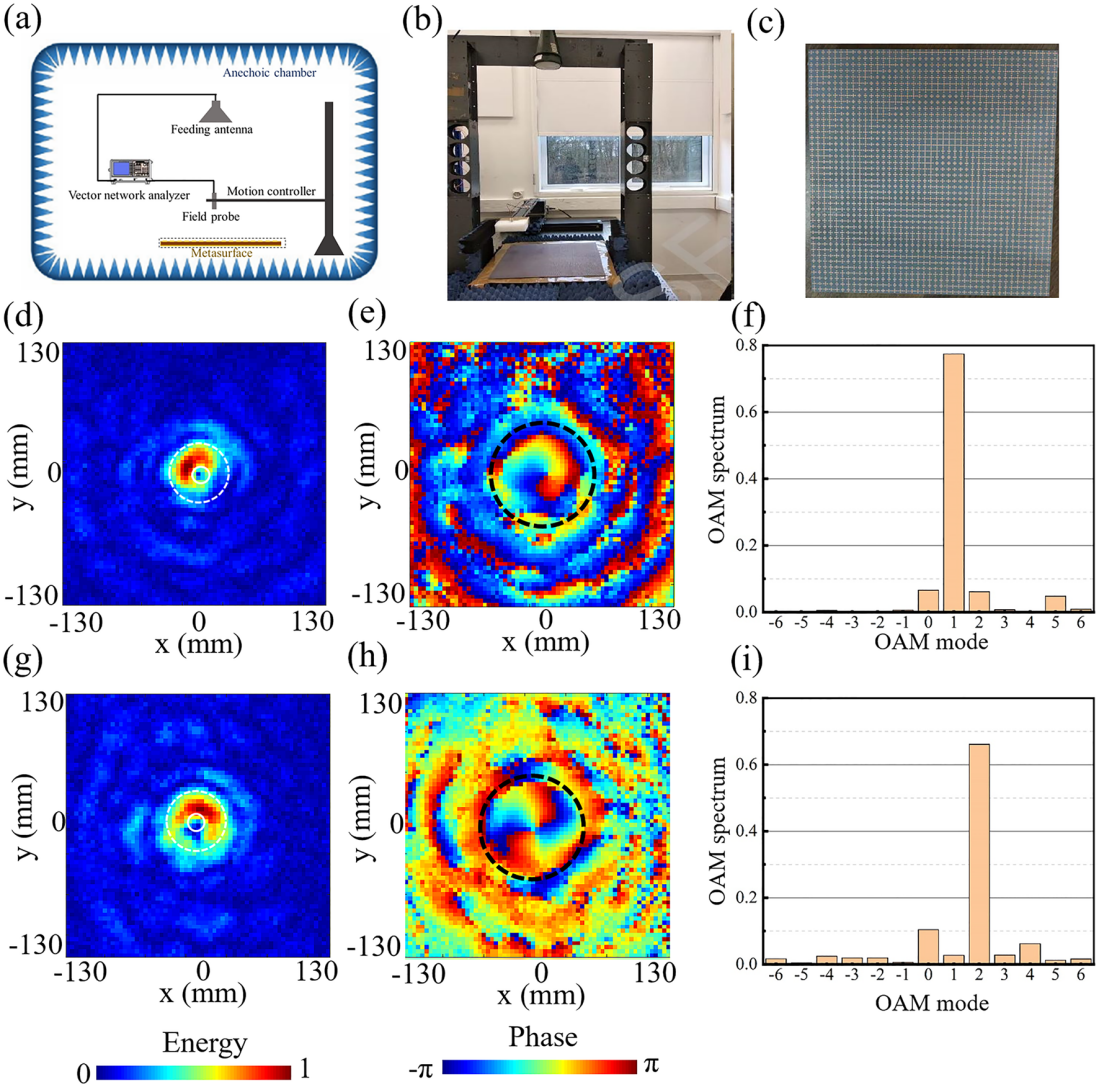
(**a**) Illustration of experimental setup. (**b**) The real measurement environment. (**c**) Photography of fabricated sample. (**d**) Measured energy distribution at *z* = 150 mm in *xoy* plane under *x*-polarized incidence, and (**e**) the corresponding phase distribution. (**f**) The measured OAM spectrum under *x*-polarized incidence. (**g**) Measured energy distribution at *z* = 150 mm in *xoy* plane under *y*-polarized incidence, and (**h**) the corresponding phase distribution. (**i**) The measured OAM spectrum under *y*-polarized incidence.

To experimentally validate the performance of the proposed dual-mode vortex beam generator, a sample is fabricated using conventional printed circuit board (PCB) techniques, as depicted by the photography in Fig. 4c. Measurements are performed using near-field scanning system, as detailed in "Methods". Figure 4d,g give the measured energy distribution in the *xoy* plane at *z* = 150 mm under *x*- and *y*-polarized incidences, respectively, where a near doughnut-shaped energy ring and the helical phase pattern distribution in Fig. 4e,h can be observed. The slight difference between measured results and simulated results shown in Fig. 3 mainly comes from the slight off-axis deviation of the incident field and the non-planar wavefronts from the feeding horn antenna across the metasurface, compared to normally incident ideal planar wavefronts considered in simulations. The simulated and measured results validate the effectiveness of generating dual-mode vortex beams utilizing our proposed 2-bit coding metasurface. In addition, the measured power distribution of different OAM modes are analyzed in the OAM purity spectrum^43^ displayed in Fig. 4f,i. Since the reflected wave is converged, only the central area encircled with black dotted line in Fig. 4e,h are used to calculate the OAM spectrum. The mode purities of the + 1 OAM order is nearly 77.4% of *x*-polarized incidence, and reaches 66.1% of *y*-polarized incidence. This discrepancy is mainly due to phase discretization losses and nonuniform incident intensity, resulting in the distribution of power in other OAM modes. To show the ultrathin property, the proposed metasurface is compared with published reflective metasurfaces which can be used to generate dual-mode OAM beams, as shown in Table. 1. The proposed metasurface not only shows the ultrathin property, but also uses only single layer substrate without air gap between the substrate and the metal ground, which provides more advantages in fabrication, installation and integration.

**Table 1:** Comparison of the proposed metasurface with referenced designs.

Ref	Frequency (GHz)	Number layers	Multiplexing style	Thickness (mm)
Meng et al.^34^	6.10	Single (with air gap)	Dual-polarization	7.8 (0.26λ_0_ at higher Freq.)
Yu et al.^38^	5.8	Single (with air gap)	Dual-band	6 (0.116λ_0_)
Iqbal et al.^44^	10.15	Bilayer	Dual-band	2 (0.1λ_0_ at higher Feq.)
Xu et al.^45^	10.5	Bilayer	Dual-polarization	5 (0.175λ_0_)
Our work	15	Single	Dual-polarization	1.5 (0.075λ_0_)

## Discussion

In summary, an efficient ultrathin measurface composed of 2-bit coding elements has been proposed for generation of dual-mode vortex beams. As building blocks, single-layer ground-backed Jerusalem cross meta-atom is introduced to achieve the phase manipulation of orthogonally polarized incident waves. By adjusting the arm length of the Jerusalem cross structures, the proposed meta-atom ensures the functionalities of near 100% reflection amplitude, 312° phase coverage, and independent control of dual linear polarizations. Then, 16 coding elements are extracted to construct the metasurface combining two coding profiles discretized from converged OAM algorithm. The measured results show good agreement with theoretical designs and simulated results, validating the effectiveness of the proposed design. The proposed ultrathin polarization-independent metamirror based vortex beam generator expands the route for manipulation of the vortex beams, and can find potential applications in wireless communication systems in microwave region.

## Methods

### Experimental measurements

Measurements are performed using near-field scanning system, and the experimental setup is shown in Fig. 4a. The real measurement environment is given in Fig. 4b. The distance between the feeding horn antenna and the metasurface is larger than 20λ to generate the desired quasi-plane waves. The fiber optic active antenna is fixed on two orthogonal translation stages, which is controlled by a motion controller to measure the electric field distribution with a step of 2 mm, and the feeding horn antenna and the fiber optic active antenna are connected to a vector network analyzer (VNA). Two measurements are implemented with and without the reflected-type metasurface to achieve the total electric field and the incident electric field respectively. Then the reflected electric field can be calculated and shown in Fig. 4c,d.
